# Influence of Backpack Carriage and Walking Speed on Muscle Synergies in Healthy Children

**DOI:** 10.3390/bioengineering11020173

**Published:** 2024-02-10

**Authors:** Giorgia Marino, Alessandro Scano, Giulia Beltrame, Cristina Brambilla, Alessandro Marazzi, Francesco Aparo, Lorenzo Molinari Tosatti, Roberto Gatti, Nicola Portinaro

**Affiliations:** 1 Physiotherapy Unit, IRCCS Humanitas Research Hospital, via Manzoni 56, Rozzano, 20098 Milan, Italy; roberto.gatti@hunimed.eu; 2 Institute of Intelligent Industrial Systems and Technologies for Advanced Manufacturing (STIIMA), Italian Council of National Research (CNR), 00187 Milan, Italy; alessandro.scano@stiima.cnr.it (A.S.); cristina.brambilla@stiima.cnr.it (C.B.); lorenzo.molinaritosatti@stiima.cnr.it (L.M.T.); 3 Residency Program in Orthopedics and Traumatology, Universitá degli Studi di Milano, 20126 Milan, Italy; beltramegiulia.gb@gmail.com (G.B.); nicola.portinaro@humanitas.it (N.P.); 4 Department of Biomedical Sciences, Humanitas University, via Rita Levi Montalcini 4, Pieve Emanuele, 20072 Milan, Italy; alessandromarazzi17@gmail.com (A.M.); francesco.aparo@st.hunimed.eu (F.A.); 5 Department of Pediatric Surgery, Fondazione IRCCS Ca’ Granda, Ospedale Maggiore Policlinico, 20122 Milan, Italy

**Keywords:** muscle synergies, locomotion, backpack carriage, gait analysis, children

## Abstract

Four to five muscle synergies account for children’s locomotion and appear to be consistent across alterations in speed and slopes. Backpack carriage induces alterations in gait kinematics in healthy children, raising questions regarding the clinical consequences related to orthopedic and neurological diseases and ergonomics. However, to support clinical decisions and characterize backpack carriage, muscle synergies can help with understanding the alterations induced in this condition at the motor control level. In this study, we investigated how children adjust the recruitment of motor patterns during locomotion, when greater muscular demands are required (backpack carriage). Twenty healthy male children underwent an instrumental gait analysis and muscle synergies extraction during three walking conditions: self-selected, fast and load conditions. In the fast condition, a reduction in the number of synergies (three to four) was needed for reconstructing the EMG signal with the same accuracy as in the other conditions (three to five). Synergies were grouped in only four clusters in the fast condition, while five clusters were needed for the self-selected condition. The right number of clusters was not clearly identified in the load condition. Speed and backpack carriage altered nearly every spatial–temporal parameter of gait, whereas kinematic alterations reflected mainly hip and pelvis adaptations. Although the synergistic patterns were consistent across conditions, indicating a similar motor pattern in different conditions, the fast condition required fewer synergies for reconstructing the EMG signal with the same level of accuracy.

## 1. Introduction

In recent decades, a growing interest has developed in understanding how backpack carriage could influence the locomotion of schoolchildren, as it could be considered to be a day-to-day challenging motor condition. Locomotion is a fundamental motor task that requires fine cyclic movements and the coordination of a vast number of variables due to the redundant degrees of freedom (DOFs) within the musculoskeletal system [1]. The central nervous system (CNS) simplifies motor control by sending motor commands that activate groups of muscles, known as synergies, that co-activate together with coordinated weighting coefficients [2,3].

Previous studies have reported that four to five muscle synergies are required during normal walking, and that they describe more than 90% of the variances in muscle activity during locomotion in healthy people [4,5,6]. Similarly, four synergies have been found in children’s locomotion, with slight differences in the muscular peak of activation that are dependent on the subjects’ ages [7]. Different walking conditions have been analyzed to assess the consistency of muscle synergies [5,8]. Specifically, temporal recruitment of synergies is altered with perturbed walking and different speeds in line with changes in the duration of gait cycle phases (i.e., stance and swing time percentage). Differently, spatial synergies appear to be similar under different environmental conditions [9,10]. Therefore, in accordance with the literature, it is possible to state that spatial synergies are robust among multiple walking conditions, but they are recruited with different timings and by different physiological structures [11]. These results reinforce the idea that the CNS relies on muscle synergies to simplify the need for motor control, allowing few descending motor pathways to achieve different motor actions [12].

Even in children, muscle synergies appear to be consistent regardless of the differences in gait kinematics [13]: higher slopes and faster speed cause increased pelvic tilt and increased hip and knee angles during early stance and late swing phases, and similar synergies are activated. The authors stated that the consistency of muscle synergies across walking conditions reveals that synergies are not simply a response to biomechanical constraints but also have a neurological origin, potentially suggesting that the CNS could preserve a synergistic pattern among biomechanical conditions [14].

An interesting analysis would be to evaluate if and how children adjust their recruitment of motor patterns during locomotion when greater muscular demands are required, as with backpackcarriage. Many studies have investigated the effects of backpack carriage on spatial–temporal parameters and reported a reduction in cadence, walking speed and swing phase, while stance and double support phase increased, mostly over 10% of body weight [15,16,17]. One study reported no changes in spatial–temporal gait asymmetry [18], whereas backpack walking did not influence spatial–temporal parameters [19]. However, a 2-D video analysis was used, and this may have simplified gait evaluation.

Variations in children’s gait kinematics at different speeds have been widely investigated [20,21], as well as the influence of backpack carriage on gait kinetics and ground reaction forces [13]. The backpack carriage generally caused increased trunk and hip flexion for counterbalancing the extra load on the back, limited pelvic rotation increased pelvic tilt for supporting the weight from the spine to the lower limbs, and an increased peak knee flexion during walking [22,23]. The forward inclination of the trunk shifts the center of mass anteriorly, making it difficult to maintain balance. Moreover, a greater anterior–posterior displacement of the center of pressure (CoP) during backpack carriage has been reported [24]. Interestingly, postural sway during backpack carriage has been poorly explored. Pau and colleagues [25] were interested in determining the impact of a backpack on balance in healthy children and reported how heavy loads increased the sway area and the CoP path length. These differences tended to decrease at older ages, probably due to the maturation of balance.

In this work, we hypothesize that kinematic alterations, in line with diminished balance, could impact the coordination of body segments during locomotion [26]. However, few studies have investigated the effects of backpack carriage on coordinated muscle activations during gait. In adults, it has been shown that the muscle activity of the quadriceps and gastrocnemii increased in response to walking with load [27,28]. One study investigated the influence of backpack carriage in children, focusing only on the trunk muscles, and found that their activity increased with increasing load [29].

To date, few studies have investigated changes in lower limb muscle activity and in muscle synergy patterns in children during backpack carriage. It has been shown that backpack carriage modifies both biomechanics and spatiotemporal gait parameters. In fact, backpack carriage is responsible for postural compensations in the trunk and lower limbs [30] that could lead to orthopedic diseases, especially in children. Indeed, backpack load has been demonstrated as being related to low back pain, which children and adolescents suffer from and that can have consequences for chronic lower back pain in adulthood [31,32]. Muscle synergy analysis provides an innovative way to describe how muscles coordinate their activations during locomotion, and it is also a valuable tool for assessing ergonomics [9]. Although muscles synergies during human walking have been extensively examined, an insight into their activation profiles and a detailed characterization of their functionality, along with changes in kinematic variables, has not been properly investigated. In this way, our understanding of the physiological mechanisms of compensations and spatiotemporal changes can be deepened, and markers of the risks of developing musculoskeletal disease can be identified. Therefore, the aim of this study was to use gait analysis and muscle synergy extraction to evaluate how fast speed and backpack carriage, common conditions of everyday life, may affect gait and muscle synergies in children.

## 2. Materials and Methods

This observational study was carried out at the Movement Analysis Laboratory of the Humanitas Research Hospital (Rozzano, Milan, Italy), in cooperation with the Italian Council of National Research (CNR, Milan, Italy). All participants signed a written informed consent before the experiment, which was conducted in accordance with the Declaration of Helsinki. The study was reviewed and approved by the Humanitas Ethical Committee.

### 2.1. Experimental Protocol

Twenty male child participants were enrolled in the Humanitas Research Hospital between February and October 2022. Participants with no musculoskeletal diseases were recruited, all aged between 8 and 12 years old. Children reporting pain, traumatic injuries, musculoskeletal or nervous system diseases were excluded.

Participants underwent a single evaluation session, which consisted of a three-dimensional gait analysis. Each recording session included three experimental conditions, preceded by a short adaptation phase to familiarize them with the task to be executed.

In the first experimental condition, the subject had to walk barefoot on a 5 m walkway at a normal, self-selected speed, (self-selected condition). In the second experimental condition, children had to walk at a fast speed (fast condition). Lastly, children were required to walk at a normal speed while carrying a backpack loaded with 12.5% of their body weight (load condition), in accordance with guidelines to prevent overloading [33,34]. The protocol had a duration of about 1 h per subject, including the sensor dressing phase.

### 2.2. Kinematic Analysis

Kinematic data during walking were obtained through an 8-camera optoelectronic system (Vicon 8 TVC system, Oxford, UK), synchronized with a 45 × 50 cm force platform (AMTI 100 MHz, Advanced Medical Technologies, Watertown, MA, USA). Before the trials, anthropometric measures were collected. Sixteen reflective markers were positioned on the anterosuperior and posterior-superior iliac spine, 1/3 of the lower lateral surface of the thigh, the lateral epicondyle of the femur,1/3 proximal of the tibia, lateral malleolus, the head of the second metatarsal and calcaneus, all in accordance with the Helen Hayes marker set and implemented in Vicon motion capture systems as the Plug-in-Gait model (PiG). The assessments were always conducted by the same two physiotherapists with experience in gait analysis. Raw marker data were low-pass filtered at 6 Hz, and force platform data were filtered at 40 Hz using a fourth-order phase-corrected Butterworth filter.

Subsequently, the data were processed using Matlab 2022. The gait cycle was defined as the period from heel contact to the subsequent heel contact on the ground. The heel-strike event was identified via the force plate data, and the subsequent step cycles were calculated using the Vicon autocorrelation pipeline before and confirmed by visual check of the vertical position of the marker on the heel [35]. To compare different gait cycles, each of them was resampled to a final length of 101 points. Spatial–temporal parameters, such as cadence (steps/min), walking speed (m/s), stride time (s), foot off (% gait cycle) and stride length (m), were calculated. Moreover, kinematic parameters of the pelvis, knee, hip, and ankle in the sagittal plane were extracted and expressed as a percentage of the gait cycle. Kinematic variables were extracted from the gait cycle on the force plate and the subsequent two gait cycles of each of the five trials in the three conditions, and then the data were averaged for each subject.

### 2.3. EMG Analysis, Synergy Extraction and Synergy Clustering

Children were instrumented with 16 s-EMG electrodes (Cometa, Milan, Italy), 8 per side, positioned according to the SENIAM guidelines [36] on the following muscles: Tibialias Anterior (TA), Gastrocnemius Lateralis (GL), Gastrocnemius Medialis (GM), Rectus Femoris (RF), Semitendinosus (ST), Biceps Femoris (BF), Adductor Magnus (ADD), Gluteus Maximus (GI). EMG data were synchronized with the kinematic data and collected simultaneously.

For this study, EMG data from a selection of barefoot gait cycles were analyzed. EMG data from both sides (right and left) were included for the recruited individuals. EMG data were sampled at 1000 Hz, bandpass filtered between 20 and 450 Hz, rectified, and then low-pass filtered at 10 Hz to achieve the envelope for each muscle. To facilitate comparisons between participants, EMG data from each muscle were normalized to their peak value and resampled at 101 samples per stride.

For each subject and experimental condition, the aligned, filtered and normalized EMG envelopes were arranged to generate the pooled matrix data to be given as input to the synergy extraction algorithm. Thus, for each subject, and for each of the three experimental modalities (self-selected, fast, and load), all movements for each trial and condition were grouped together to be used as input for synergy extraction. Five to seven gait cycles were used for each subject in each experimental condition.

**Figure 1:**
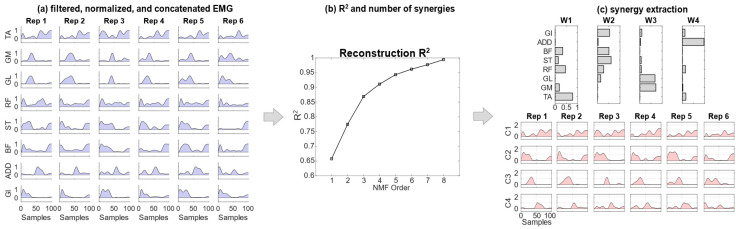
A scheme of the EMG workflow: (**a**) EMG signals are filtered, rectified and normalized. All the repetitions are concatenated; (**b**) the reconstruction R^2^ is computed and the number of synergies achieving at least R^2^ = 0.90 is selected; (**c**) synergies are extracted. Rep indicates the number of the strides considered for the analysis.

Each EMG pooled matrix had dimensionality [(n_samples_) × n_tasks_] × [n_muscles_], concatenating together data from the repetitions of strides for each experimental condition [29]. In detail, this involved n_tasks_ = 5–7 (number of strides), n_muscles_ = 8 and n_samples_ = 101. Then, spatial synergies were extracted using the non-negative matrix factorization (NMF) algorithm [30]. The NMF decomposed the EMG data matrix into the product of two matrices, the first one representing time-invariant synergies (*w_i_*), and the second one representing time-varying activation coefficients for each synergy (*c_i_*), as in Equation (1):

(1) $$EMG\left(t\right)=\sum_{i=1}^{N}c_{i}\left(t\right)w_{i}$$

where, for each of the recorded muscles, *EMG*(*t*) represents the EMG data at time *t* and *N* is the total number of extracted spatial synergies (Figure 1) [37].

Thus, for each decomposition, each spatial synergy was coupled with a set of *n* coefficients (dimensionality *n* equal to the total number of time samples of all included movements n_tasks_×n_samples_), and each set had dimensionality *N* equal to the number of extracted synergies.

The number of extracted synergies (order of the factorization), *r*, given as input to the NMF algorithm, was chosen increasingly from 1 to 8 (the maximum number of muscles in each leg). For each *r*, the NMF algorithm was applied 100 times to avoid local minima, while the repetition accounting for the highest fraction of the total variation of the signal explained by the synergy reconstruction was chosen as the factorization of order *r*.

The number of synergies, *N*, was then chosen as the minimum *r* explaining at least 90% of the data variation, quantified by the reconstruction R^2^ defined as 1—SSE/SST, where SSE is the sum of the squared residuals and SST is the sum of the squared differences with the mean EMG vector [38].

The structure of synergies (i.e., the relative weights of muscles in each synergy of W) was compared using a K-means cluster analysis. The NMF algorithm estimates synergies that can describe variance in muscle activity for each individual, but we aimed at a robust method to compare the structure of synergies across individuals. To determine common synergies between individuals, we used a K-means cluster analysis, which identifies clusters of synergies that are similar across individuals and returns the average weights of common synergies. For a given number of synergies, K-means cluster analysis identifies *n* clusters that minimize the sum over all clusters of the squared Euclidean distance from each individual’s synergies [39]. The outputs of the analysis were the cluster centroids, representing the average weights of common synergies across individuals. We used the K-means cluster analysis to identify the average synergies for the individuals in each of the experimental conditions (self-selected, fast and load). Since there is not an unequivocal criterion for the choice of the optimal number of clusters, the clustering procedure was repeated, setting the number of clusters from 1 to 6 clusters in order to compute the precision curve in function of the number of clusters. We quantified the most suitable number of mean clusters to extract for each condition by selecting a predefined threshold for the increase in similarity of the synergies belonging to the same cluster (intra-cluster similarity) when increasing clustering order. When the appropriate number of clusters was reached, we expected that the maximum uniformity of the elements in the clusters was almost reached; increasing the clustering order would minimally affect clustering precision, indicating that no more clusters were needed. This process allowed us to select the correct number of clusters to appropriately describe the number of mean synergies available for each of the experimental conditions (self-selected, fast, load) and characterize the dimensionality of the control. The number of clusters was chosen as the highest number that gives an increase in the mean intra-cluster similarity (computed as the similarity of the synergies belonging to the same cluster) > 0.025.

### 2.4. Outcome Measures and Statistics

Statistical analysis was performed using IBM SPSS Statistics version 28.0.1. The level of significance was set at α = 0.05. Spatial–temporal and kinematic measurements were checked for normality using the Shapiro–Wilk test. Subsequently, data were analyzed using a one-way analysis of variance (ANOVA) for repeated measures. In cases of significant interactions, between-condition differences were detected using Bonferroni post hoc analysis.

When comparing the number of extracted synergies between self-selected, fast and load conditions, the data were not normally distributed. Thus, we employed a non-parametric Kruskal–Wallis test. A post hoc test was used for multiple comparisons across the different modes. The intra-cluster similarity of the spatial synergies (a measure of synergy consistency across participants) was computed as the scalar product between all the pairs of synergies in the cluster, while the intra-cluster similarity of the temporal coefficients was computed as the correlation coefficient between all the pairs of temporal coefficients in the cluster. The intra-cluster similarity of the spatial synergies and of the temporal recruitment in self-selected, fast and load modes were compared with the Kruskal–Wallis test and a post-hoc test was used for multiple comparisons. For all the tests, the significance level was α = 0.05. Finally, the clusters of each condition were matched with the clusters of the other conditions, and inter-condition similarity was computed between the matched clusters. As for the intra-cluster similarity, inter-condition similarity was computed as the scalar product between matched mean spatial synergies and as the correlation coefficient between the corresponding mean temporal coefficients.

## 3. Results

Twenty healthy young male children with no neurological or orthopedic pathologies participated in the study (Table 1).

### 3.1. Spatial–Temporal and Kinematic Parameters

Most of the spatial–temporal parameters showed significant differences when comparing the three conditions (Table 2). As expected, walking speed and cadence were greater in the fast condition when compared to the self-selected and load ones, whereas stride time was significantly reduced (Self-Selected vs. Fast: MD: 0.23, *p* < 0.001, CI_95_: 0.16, 0.30; Load vs. Fast: MD: 0.17, *p* < 0.001, CI_95_: 0.08, 0.25). Moreover, a post hoc analysis revealed greater stride length in the fast condition both when compared to the self-selected (MD: 0.16, *p* < 0.001, CI_95_: 0.09, 0.23) and load (MD: 0.14, *p* < 0.001, CI_95_: 0.09, 0.19) conditions.

**Figure 2:**
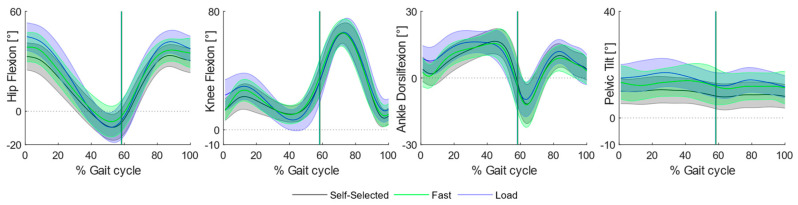
Sagittal plane angular kinematics during overground walking. Ensemble means (thick lines) and standard deviation (shaded regions) across participants of hip and knee flexion, ankle dorsiflexion and pelvic tilt across the three experimental conditions: self-selected (black curve), fast (blue curve) and load (green curve) conditions.

Increasing the load and fast speed mainly caused variations at the proximal joints, whereas, at the level of the knee joint, variations appeared mostly while walking at faster speed when compared to self-selected locomotion and backpack carriage (Figure 2).

All knee peak angles increased in favor of the fast condition: children required greater knee flexion at initial contact when compared to the self-selected (MD: 9.53, *p* < 0.001, CI_95_: 5.00, 14.05) and load (MD: 10.32, *p* < 0.001, CI_95_: 6.07, 14.56) conditions, as well as an incremented knee extension (Fast vs. Self-Selected: MD: −3.51, *p* < 0.001, CI_95_: −5.49, −1.54; Fast vs. Load: MD: −2.99, *p* < 0.001, CI_95_: −4.13, −1.84) at stance in the fast condition.

Most of the differences were found at the level of hip and pelvis peak angles. Specifically, children required a greater hip flexion angle at initial contact both while walking at fast speed (Hip: MD: 11.01 *p* < 0.001 CI_95_: 6.30, 15.72) and while carrying a backpack (Hip: MD: 5.15, *p* < 0.001, CI_95_: 2.86, 7.45). Moreover, the fast condition required greater hip flexion during swing both when compared to self-selected (MD: 7.35, *p* < 0.001, CI_95_: 4.62, 10.09) and load (MD: 3.62, *p*: 0.017, CI_95_: 0.56, 6.69) conditions, as well as an incremented hip angle at swing observed during backpack carriage (MD: 3.73, *p* < 0.001, CI_95_: 1.52, 5.94). Overall, walking at fast speed required a significantly greater hip range of motion at stance when compared to self-selected (MD: 12.59, *p* < 0.001, CI_95_: 7.72, 17.95) and load (MD: 9.06, *p* < 0.001, CI_95_: 4.39, 13.72) conditions, succeeded by greater values during backpack carriage with respect to the self-selected condition (MD: 3.53, *p*: 0.025, CI_95_: 0.38, 6.68).

At the level of the pelvis, major differences were found regarding the maximum pelvic tilt angle: children walked with less anterior pelvic tilt at self-selected speed when compared to backpack carriage (MD: −4.95, *p* < 0.001, CI_95_: −7.81, −2.09) and fast walking (MD: −5.54, *p* < 0.001, CI_95_: −7.78, −3.31).

No substantial variations were found at the level of the ankle, except for a greater peak dorsiflexion angle in the fast condition when compared to self-selected (MD: 4.27, *p*: <0.001, CI_95_: 1.88, 6.67) and load (MD: 5.88, *p*: <0.001, CI_95_: 2.36, 9.40) conditions.

### 3.2. Synergy Analysis

#### 3.2.1. Number of Extracted Synergies

**Figure 3:**
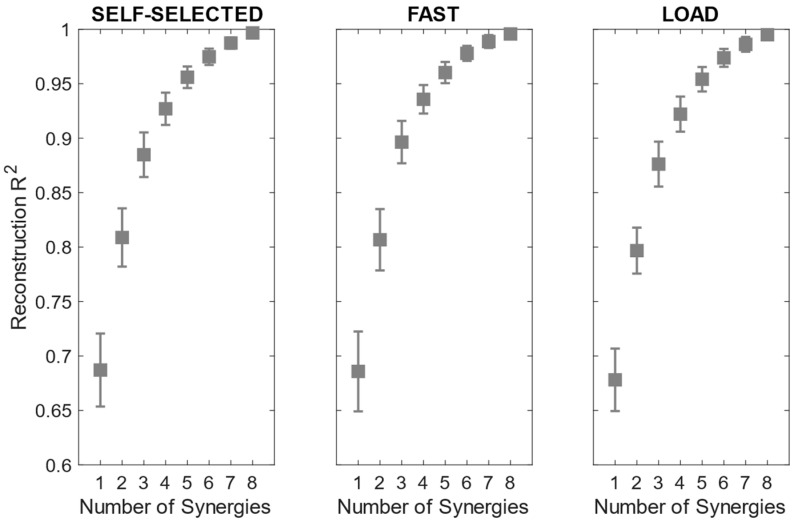
Mean and standard deviation of the reconstruction R^2^ across participants in the self-selected, fast and load conditions.

The reconstruction R^2^ curves for all the participants in each condition are portrayed in Figure 3. The number of extracted synergies was chosen as the minimum number needed to explain at least 90% of the data variation [40].

The number of extracted synergies ranged from three to five in the self-selected and load conditions and from three to four in the fast condition. The mean number of extracted synergies was significantly more reduced in the fast condition than in the load condition (Table 3).

#### 3.2.2. Synergy Clustering

**Figure 4:**
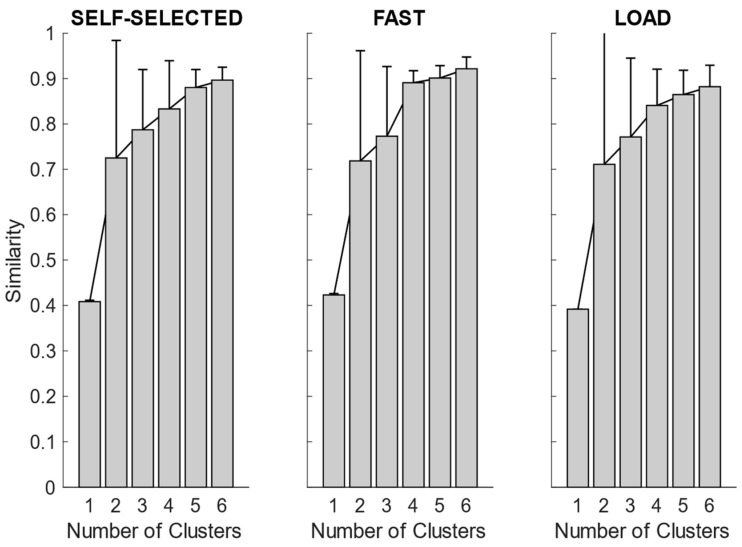
Synergy similarity when increasing the number of clusters. In the self-selected condition, and partially in the load condition, increasing the number of clusters from four to five improved the quality of the solutions. In the fast condition, only 4 clusters were needed, and increasing further the number of clusters did not achieve any advantage.

After synergy extraction, for each of the experimental conditions, we quantified the mean synergies via K-means clustering. In Figure 4, we showed the intra-cluster similarity achieved in the self-selected, fast and load conditions when increasing the number of clusters from one to six. The chosen number of clusters for the self-selected condition was five, since the increase in intra-cluster similarity was 0.047 between four and five clusters and 0.016 between five and six clusters, and therefore below the defined similarity threshold (adding more clusters led to no substantial improvement in synergy grouping). Four clusters were identified for the fast condition, as the increase in intra-cluster similarity between four and five clusters was 0.010. For the load condition, the right number of clusters was not clearly identified because the increase in intra-cluster similarity between four and five clusters was 0.024, just slightly below the predefined threshold. Therefore, we reported the results for both the four and five clusters.

**Figure 5:**
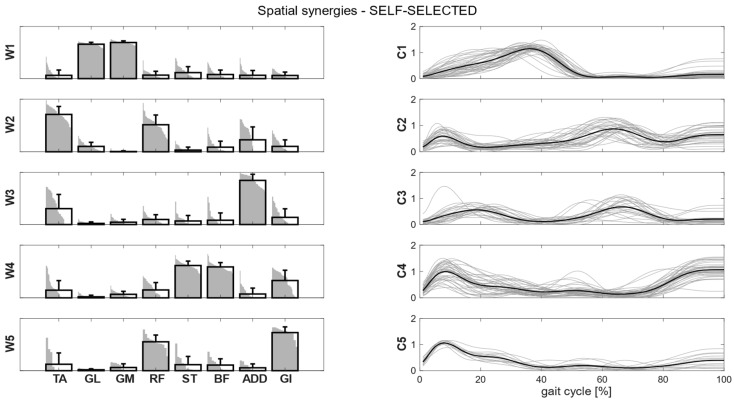
Composition (synergy structure, **left column**) and temporal coefficients (**right column**) for matched synergies for the identified clusters, in the self-selected condition. Synergies represent the activation level of each muscle, while the temporal coefficients indicate the timings of each synergy during the gait cycle. Bold black bars are the mean synergy of the cluster, while the gray bars represent all the synergies belonging to the cluster. Bold black lines represent the mean temporal coefficient of the cluster, while the gray lines represent all the temporal coefficients belonging to the cluster.

**Figure 6:**
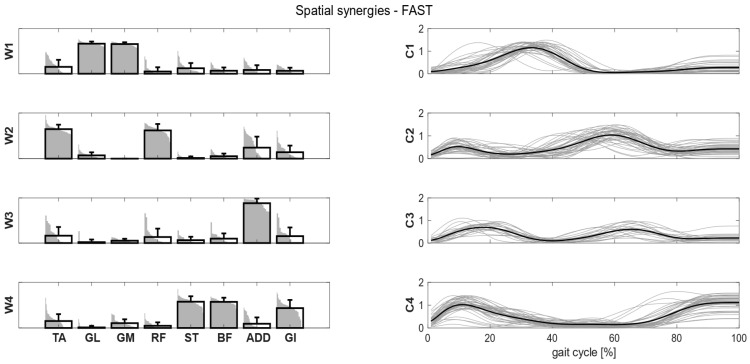
Composition (synergy structure, **left column**) and temporal coefficients (**right column**) for matched synergies for the identified clusters in the fast condition. Synergies represent the activation level of each muscle, while the temporal coefficients indicate the timings of each synergy during the gait cycle. Bold black bars represent the mean synergy of the cluster, while the gray bars represent all the synergies belonging to the cluster. Bold black lines are the mean temporal coefficient of the cluster, while the gray lines represent all the temporal coefficients belonging to the cluster.

**Figure 7:**
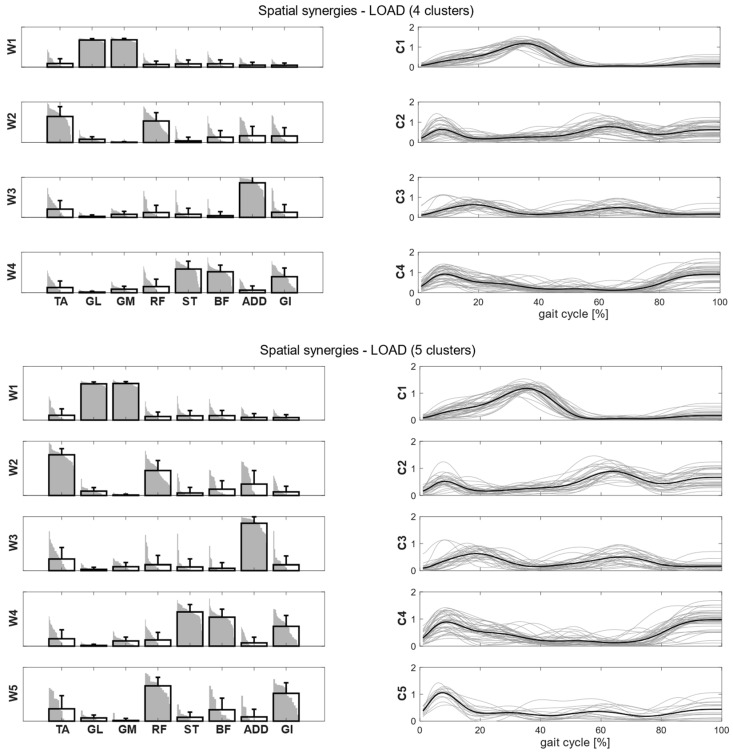
Composition (synergy structure, **left column**) and temporal coefficients (**right column**) for matched synergies for the identified clusters in the load condition in two cases: four clusters (**upper panel**) and five clusters (**lower panel**). Synergies represent the activation level of each muscle, while the temporal coefficients indicate the timings of each synergy during the gait cycle. Bold black bars represent the mean synergy of the cluster, while the gray bars represent all the synergies belonging to the cluster. Bold black lines are the mean temporal coefficient of the cluster, while the gray lines represent all the temporal coefficients belonging to the cluster.

Clusters found in the self-selected conditions represented the main features of human walking (Figure 5). In the fast condition, four clusters were found and matched with the first four synergies of the self-selected condition (Figure 6). In the load condition, solutions with both four and five clusters were analyzed and matched with the other walking conditions (Figure 7). Each cluster can be associated with a biomechanical functionality within the gait cycle, depending on the muscles recruited and the timings of activation. W1 (spatial synergy 1) was characterized by the strong activation of GL and GM, and it was activated in the late stance. W2 recruits mainly the TA and the RF, and it was activated at the beginning of the stance and during the swing phase, contributing to knee extension at swing (RF) and to the correct foot positioning at heel strike (TA). W3 was characterized by a strong activation of ADD during double support phases. W4 appears to represent the activation of the hamstrings (ST and BF) with a small contribution of TA and GI at the beginning and end of the gait cycle, whereas W5 recruits the RF, the GI and, to a lesser extent, the hamstrings and the ADD at the beginning of the stance.

#### 3.2.3. Comparison between Conditions

Intra-cluster similarity is >0.76 for the spatial synergies, indicating strong synergy consistency between participants (Table 4). Intra-cluster similarity was higher than the random similarity obtained by randomly pairing synergies (0.41 for self-selected, 0.42 for fast, 0.39 for load). W1 was the most consistent and recognizable synergy that was shared across participants. Synergies and temporal coefficients were more consistent in the fast condition than in the self-selected and load conditions in W2 and W4 (*p* < 0.001) and less consistent in W1 (*p* < 0.001). W2 and W4 were more consistent in the self-selected condition than in the load condition (*p* < 0.001). The inter-condition similarity indicated that both spatial and temporal patterns were preserved in all the conditions. In particular, W1 showed the greatest similarity across all the conditions. Spatial synergies were more similar between the self-selected and fast conditions, while the temporal coefficients were more similar between self-selected and load conditions.

Intra-cluster similarity increased for both spatial synergies (>0.80) and temporal coefficients (>0.44), as one cluster was added in the load condition (Table 5). However, synergies and temporal coefficients were more consistent in the fast condition than in the load condition in W2 and W4 (*p* < 0.001), and less consistent in W1 (*p* < 0.001). W4 and W5 were more consistent in the self-selected condition than in the load condition (*p* < 0.001). The inter-condition similarity indicated that both spatial and temporal patterns were preserved in all the conditions, and inter-condition similarity increased with five clusters. W1 showed the greatest similarity across all the conditions. Spatial synergies were more similar between self-selected and fast conditions, while the temporal coefficients were more similar between self-selected and load conditions.

## 4. Discussion

Backpack carriage in children is a common daily condition that has demonstrably induced changes in the biomechanics of the trunk and lower limbs [30], appearing to lead to orthopedic diseases in children [31]. Although the impact of backpack carriage on children’s locomotion has been previously investigated [15,16,17,18,19], little is known about how lower limb muscles coordinate their activations during locomotion when an extra load is added. The aim of this study was to examine the effect of three walking conditions on muscle synergies in young healthy children during barefoot walking. We analyzed barefoot walking in order to use the Vicon inverse kinematic routines with more precision with respect to any other non-barefoot condition. In this way, these results could be used in future work as reference data for the evaluation of disability in children, as their motor capabilities can be evaluated more correctly in barefoot conditions. Results showed that a lesser number of synergies was required in the fast condition for reconstructing the EMG envelope with the same level of accuracy. Moreover, fewer clusters were needed to group synergies across participants in the fast condition with respect to the self-selected one, while the number of clusters for the load condition was not clearly defined. In general, synergies in the fast condition showed greater consistency than those in the self-selected and load conditions, indicating that walking speed influenced the variability of synergies across children and that high speed tends to uniform muscular patterns.

Walking speed affected nearly every aspect of the spatial–temporal and kinematic gait variables, whereas the results from backpack carriage partially aligned with previous studies. A heavy load caused a significant but limited increase in speed, with no differences in stride length when compared to the self-selected condition. However, effects on walking speed have not been consistent between studies: Chow and colleagues [16] reported a decrease in gait speed, whereas no significant changes were observed in other studies [23]. These findings could hinder different behavioral responses to an external load, possibly due to children’s locomotion maturity, but further research is needed. It should be noted that, to maintain almost the same walking speed, when pelvic rotation is limited due to backpack carriage, children might prefer to increase hip excursion in the sagittal plane (i.e., greater step length) or increment step frequency, as our results highlight [41].

Muscular patterns and temporal activations were consistent across conditions, showing great similarities, and it is possible to speculate about their specific functions in the gait cycle. W1 was the most consistent synergy across conditions and showed the lowest variability. This synergy was active in counteracting the external dorsiflexion moment acting on the ankle from mid-stance until pre-swing, as well as during the push-off phase, providing the propulsion of the center of mass with the GL and GM [42]. W2 contributed to the weight acceptance at loading response by activating the TA, to prevent the foot slap and the RF, to control the external flexion moment acting on the knee. Moreover, W2 appeared to contribute to leg reposition during pre-swing and swing (i.e., rectus femoris), while TA appears to be essential during mid swing in ensuring foot clearance.

W3 appeared to provide mediolateral stability in the double support phases of the gait cycle, assisting in postural control during the mediolateral transfer of body weight with the activation of the ADD. Moreover, the contribution of the tibialis anterior to W3 is essential in the loading response phase, helping to control the pronation movements acting at the level of the subtalar joint. W4 appears to have a role in preventing the advancement of body segments and decelerating the leg during the terminal swing, activating the hamstrings and, to a lesser extent, the GI. Moreover, the ST, BF and GI roles during double limb support are to avoid the forward movement of the trunk and pelvis, which would be provoked by their inertial moment at the contact of the foot [43].

Finally, W5 appears to be related to body support and weight acceptance both at the beginning and end of the gait cycle. This synergy occurred in the same phase of the gait cycle as W4, and it was present only when five clusters were considered. It appears to be a merged synergy that encapsulates muscle contributions from W2 and W4. In the self-selected condition, W5 was expressed by RF and GI, whereas backpack carriage also required ST and BF contributions. Rectus femoris and gluteus are fundamental to controlling knee and hip flexion at loading response, provoked by the vertical component of the body inertial moment when the foot stops on the ground at the beginning of the gait cycle. In line with previous studies, reporting an increase in a stance and a double support time [17], the role of W5 might be a means of effectively managing the load during gait.

Regarding the kinematics, in line with synergistic findings, children appeared to commonly perform proximal adaptations to handle different walking conditions. Both backpack carriage and fast speed, indeed, likely caused an increased pelvic anteversion during the gait cycle, along with greater hip flexion angles. This adaptation in the fast condition can be assumed to be necessary in accomplishing a longer stride (as a consequence of faster speed), whereas during backpack carriage it might be a postural adjustment to counteract the extra loading on the back [22,23].

Kinematic results showed a greater knee flexion angle at stance while walking at fast speed [21]. One could speculate that the RF contribution should be greater in the fast condition; however, the emergence of W5 may not be required during the fast condition because of the reduction in the duration of stance (as a function of speed) and the higher muscle co-contraction required at increased velocity [44]. This is in line with previous studies in which, by increasing the speed of locomotion, fewer muscular synergies were extracted and the original EMG signal could be reconstructed more efficiently [45]. Moreover, walking faster requires greater horizontal forces to forward the propulsion of the center of mass [46]. Along with a reduction in the stance phase in line with speed increase [44], we expect a greater contribution of muscles acting to decelerate the trunk and lower limbs [46] rather than a contribution of muscles acting to prevent shock absorption and leg stability [47]. Although this aspect could explain the disappearance of W5 in the fast condition, we did not focus on the center of mass displacement, and thus additional investigations are needed to support this hypothesis.

Another interesting aspect was the decreased variability across participants at fast speed, indicating that faster walking speed tends to uniformize the muscular patterns thanks to greater and more defined muscle activity [44]. This probably occurs since fast walking is executed mainly in feed-forward control, activating more stereotyped muscle synergies [48]. In contrast, normal walking, especially load walking, may involve feedback circuitry slightly increasing the control variability. Backpack carriage, indeed, increased the mean number of intra-subjects-extracted synergies with respect to the fast condition, but the effect on the variability is not clear. In fact, in the load condition, more synergies were needed for reconstructing the EMG signal with the same level of accuracy as the fast one, but the number of clusters needed to group the synergies did not increase. The effect of variability in the load condition deserved to be punctuated: though walking speed slightly differed between self-selected and load conditions, it resulted in being significantly greater. However, no randomization in the three walking paradigms was undertaken, and this might have impacted the walking speed results. Thus, the emergence of a not-predominant W5 during backpack carriage might be related to inter-subject differences in speed, which could have altered synergy expression, as stated before. As previously stated, backpack influence on walking speed is not clear, and these discrepancies might hide different behavioral responses to backpack carriage. From this point of view, it could be speculated that the presence of a non-defined number of clusters to group synergies in the load condition might be related not only to differences in speed but to different coordination actions of muscles during gait. An interesting analysis would be differentiating backpack carriage over different age groups of children to better understand to what extent locomotion maturity affects backpack carriage.

In line with the muscular pattern, walking with a backpack required increased hip flexion during the entire gait cycle to shift forward the center of gravity [19,38] to counterbalance the extension moment caused by backpack carriage. Moreover, a load greater than 10% BW induced significant changes in pelvic anteversion, in line with what was stated by Devroey and colleagues [39], whose studies aimed at investigating changes in trunk posture related to backpack weight and position. To this extent, W5 contribution of ST and BF in the load condition might be necessary to better control the forward inclination of the trunk and pelvis in order to better manage balance at loading response. Similar synergistic patterns were found in Bejerano et al. [34] in which four patterns, related to weight acceptance, push-off, trunk balance and leg deceleration, were identified in both linear and rectilinear walking trajectories. W1 is required to provide propulsion during the push-off phase, whereas W2 contributes mainly to repositioning the lower limb during the gait cycle. To this extent, W4 and W5 contributions could respectively be related to the control of the horizontal inertial moment acting on the lower limb (i.e., leg deceleration during swing and hip extension) and to counteracting the vertical forces acting during the weight acceptance phase. An interesting aspect to note is how children adopt a muscle coordination pattern similar to the self-selected condition, although backpack load induced alterations in the kinematics aspects of gait, along with increased postural instability.

There are several consequences related to the results of this work. First, our work may be a first step towards understanding the ergonomics of backpack carriage in terms of alteration of motor control. Even though our kinematic findings are in line with previous literature, we could not find any study investigating muscle synergies in backpack carriage. Our study showed that, in terms of motor control, the backpack carriage is a “middle” condition between normal walking and fast walking in terms of the number and composition of synergies. It is well established that muscle synergies are altered in children suffering from cerebral palsy [49]. Our findings can be used as reference data to evaluate muscle synergies in patients with cerebral palsy across many applications, including when considering the evaluation of the effects of surgical interventions, altered walking conditions (including assisted devices, orthosis, backpack carriage and others), or to analyze how they affect motor control. Previous studies have reported how changes in speed and a support base reduction during walking significantly alter the number of extracted synergies in cerebral palsy, but not in typically developed children [50]. To this extent, the role of external load on cerebral palsy could be further analyzed, as well as its implications for muscle coordination. This will help in designing proper rehabilitation courses and calibrating the best therapeutic approaches. Third, the data gathered in this experiment will also be used to improve the application of algorithms for interpreting motor control by applying the novel mixed-matrix factorization [51] to connect neural variables (i.e., muscle synergies) with task variables (i.e., kinematics and kinetics) in the hope of providing novel instruments for assessing pathological gait.

This work has some limitations. At first, it should be remarked that the use of 16 EMG channels (eight per limb) while being in line with the standard used in reference articles in the literature [52] is still limiting considering the large number of muscles in the lower limbs. Previous works have warned about the use of a restricted number of channels, suggesting that some modules might be missed or misinterpreted [53,54]. Future work should also investigate kinematics and muscle synergies at the trunk level.

Secondly, other methodological approaches (including synergy extraction algorithms and EMG normalization) could be considered to compare results. Other EMG normalization methods could be considered, including normalizing to the average amplitude for each channel, normalizing to achieve unit variance, normalizing each stride by the maximum EMG, or other methods. The method of normalization used in this study was selected based on a recent review [55] that revealed that it is the most commonly used method for analyzing data in cerebral palsy experimental trials. Moreover, some studies focused on the effects of the normalization and concluded that no main effect of normalization was found when EMG data were either not normalized, normalized to their own maximum, or normalized to the pre-treatment maximum [56,57]. The application of the protocol used in this study regarding neurological patients’ performances might benefit from fine-tuned recordings for the matching of the reference database to the peculiar features of motor impairment (e.g., reduced range of motion, jerky movements, lack of repeatability), which were not investigated in this study. The extracted synergies might be evaluated in the light of novel concepts, such as kinematic–muscular synergies [51], to directly relate muscle activations to kinematic outputs.

Moreover, exploring the influence of different backpack weights on muscle synergies across different age groups could enhance our understanding of how the central nervous system adapts muscle coordination in more challenging conditions. Additionally, the cohort of enrolled children in this study is limited in number, comprising solely male subjects. Future work may include a wider cohort of participants that is uniformly distributed between genders.

## 5. Conclusions

In this study, we investigated the effects of walking speed and backpack carriage on the extraction of muscle synergies. Our results showed that fewer synergies were required in the fast condition for reconstructing the EMG signal with the same level of accuracy. Moreover, fewer clusters were needed to group synergies across participants in the fast condition with respect to the self-selected and load conditions. In general, synergies in all the conditions showed strong intra-cluster similarities and could be related to biomechanical phases of the gait cycle. Both spatial synergies and temporal coefficients were consistent across different conditions, showing that the same basic motor patterns characterize the motor control in each condition. However, it seems that walking synergies are also dependent on the biomechanical request. Our results could be used as a benchmark of muscle synergies extracted from children walking under different conditions.

## Figures and Tables

**Table 1:** Baseline characteristics of the study participants (*n* = 20). Data are shown as mean and standard deviation (SD).

	Healthy Children
Age (y)	8.7 ± 1.1
Weight (kg)	29.6 ± 7.2
Height (cm)	133.3 ± 9.5
Backpack Load (kg)	3.5 ± 0.7

**Table 2:** Results of spatial–temporal parameters of gait for self-selected, fast and load walking conditions. Data are shown as mean and standard deviation.

	Self-Selected	Fast	Load	*p*-Value
Cadence (steps/min)	119.3 ± 10.7	154.3 ± 16.3 **	127.7 ± 13.1 *^#^	<0.001
Walking speed (m/s)	1.1 ± 0.1	1.6 ± 0.2 **	1.2 ± 0.2 *^#^	0.004
Stride time (s)	1.0 ± 0.1	0.8 ± 0.1 **	1.0 ± 0.1 ^#^	<0.001
Foot off (%)	58.7 ± 1.6	57.5 ± 2.3	58.1 ± 1.5	0.333
Stride Length (m)	1.1 ± 0.1	1.3 ± 0.1 **	1.1 ± 0.1 ^#^	<0.001

* *p* < 0.05 when compared to self-selected; ** *p* < 0.001 when compared to self-selected; ^#^ *p* < 0.001 when compared to fast.

**Table 3:** Comparison of the number of synergies across conditions. The table reports the mean and standard deviation across participants of the number of extracted synergies with R^2^ > 0.90. Standard deviations are indicated in brackets. Statistical tests are reported, and significant *p* are shown in bold. S = Self-Selected, F = Fast, L = Load.

	Self-Selected Mean	Fast Mean	Load Mean	S vs. F *p*	S vs. L *p*	F vs. L *p*
Number of synergies	3.81 (0.52)	3.62 (0.49)	3.95 (0.40)	0.23	0.42	**0.01**

**Table 4:** Mean intra-cluster similarity (upper panel) and inter-condition similarity (lower panel) are reported for each cluster and for both spatial synergies and temporal coefficients, considering four clusters for the load condition. The mean intra-cluster similarity is the mean similarity computed with all the possible pairings of synergies (or temporal coefficients) belonging to that cluster. Standard deviations are reported in brackets. The inter-condition similarity is computed as the similarity between matched clusters of different conditions. S = Self-Selected, F = Fast, L = Load.

	Mean Intra-Cluster Similarity					
	Spatial Synergies			Temporal Coefficients		
	Self-Selected	Fast	Load	Self-Selected	Fast	Load
W1	0.95 (0.04)	0.93 (0.04)	0.95 (0.04)	0.86 (0.14)	0.80 (0.22)	0.88 (0.12)
W2	0.84 (0.10)	0.88 (0.10)	0.76 (0.18)	0.54 (0.29)	0.59 (0.32)	0.41 (0.33)
W3	0.84 (0.12)	0.86 (0.10)	0.83 (0.15)	0.52 (0.33)	0.56 (0.28)	0.43 (0.36)
W4	0.88 (0.09)	0.89 (0.10)	0.81 (0.15)	0.72 (0.22)	0.74 (0.23)	0.59 (0.33)
W5	0.87 (0.09)	-	-	0.82 (0.13)	-	-
	**Inter-Condition Similarity**					
	**Spatial Synergies**			**Temporal Coefficients**		
	**S vs. F**	**S vs. L**	**F vs. L**	**S vs. F**	**S vs. L**	**F vs. L**
W1	0.93	0.95	0.94	0.97	0.99	0.98
W2	0.86	0.80	0.82	0.78	0.98	0.70
W3	0.84	0.83	0.84	0.95	0.91	0.98
W4	0.87	0.84	0.85	0.95	0.99	0.97

**Table 5:** Mean intra-cluster similarity (upper panel) and inter-condition similarity (lower panel) are reported for each cluster and for both spatial synergies and temporal coefficients, considering five clusters for the load condition. The mean intra-cluster similarity is the mean similarity computed with all the possible pairings of synergies (or temporal coefficients) belonging to that cluster. Standard deviations are reported in brackets. The inter-condition similarity is computed as the similarity between matched clusters of different conditions. S = Self-Selected, F = Fast, L = Load.

	Mean Intra-Cluster Similarity					
	Spatial Synergies			Temporal Coefficients		
	Self-Selected	Fast	Load	Self-Selected	Fast	Load
W1	0.95 (0.04)	0.93 (0.04)	0.95 (0.04)	0.86 (0.14)	0.80 (0.22)	0.88 (0.12)
W2	0.84 (0.10)	0.88 (0.10)	0.84 (0.10)	0.54 (0.29)	0.59 (0.32)	0.56 (0.26)
W3	0.84 (0.12)	0.86 (0.10)	0.85 (0.13)	0.52 (0.33)	0.56 (0.28)	0.44 (0.36)
W4	0.88 (0.09)	0.89 (0.10)	0.86 (0.11)	0.72 (0.22)	0.74 (0.23)	0.62 (0.32)
W5	0.87 (0.09)	-	0.80 (0.12)	0.82 (0.13)	-	0.54 (0.31)
	**Inter-Condition Similarity**					
	**Spatial Synergies**			**Temporal Coefficients**		
	**S vs. F**	**S vs. L**	**F vs. L**	**S vs. F**	**S vs. L**	**F vs. L**
W1	0.93	0.95	0.94	0.97	0.99	0.98
W2	0.86	0.84	0.84	0.78	0.99	0.74
W3	0.84	0.84	0.85	0.95	0.93	0.98
W4	0.87	0.87	0.87	0.95	0.99	0.98
W5	-	0.80	-	-	0.89	-

## Data Availability

The datasets used and analyzed during the current study are available from the corresponding author upon reasonable request.

## Abbreviations

DOFs	degrees of freedom
CNS	central nervous system
CoP	center of pressure
PiG	plug-in-gait
EMG	electromyography
TA	tibialis anterior
GL	gastrocnemius lateralis
GM	gastrocnemius medialis
RF	rectus femoris
ST	semitendinosus
BF	biceps femoris
ADD	Adductor magnus
GI	Gluteus maximus
NMF	non-negative matrix factorization
SSE	sum of the squared residuals
SST	sum of the squared differences
ANOVA	analysis of variance
MD	mean difference
CI_95_	confidence interval 95%
W1-W5	synergy clusters
